# Modeling of Ion Agglomeration in Magnesium Electrolytes and its Impacts on Battery Performance

**DOI:** 10.1002/cssc.202001034

**Published:** 2020-06-29

**Authors:** Janina Drews, Timo Danner, Piotr Jankowski, Tejs Vegge, Juan Maria García Lastra, Runyu Liu, Zhirong Zhao‐Karger, Maximilian Fichtner, Arnulf Latz

**Affiliations:** ^1^ Institute of Engineering Thermodynamics, Computational Electrochemistry German Aerospace Center (DLR) Pfaffenwaldring 38–40 70569 Stuttgart Germany; ^2^ Helmholtz Institute Ulm (HIU) Helmholtzstr. 11 89081 Ulm Germany; ^3^ Department of Energy Conversion and Storage Technical University of Denmark (DTU) Anker Engelunds Vej 2800 Kgs. Lyngby Denmark; ^4^ Institute of Nanotechnology (INT) Karlsruhe Institute of Technology (KIT) Hermann-von-Helmholtz-Platz 1 76344 Eggenstein-Leopoldshafen Germany; ^5^ Institute of Electrochemistry Ulm University (UUlm) Albert-Einstein-Allee 47 89081 Ulm Germany

**Keywords:** batteries, computational chemistry, electrolytes, ion aggregation, magnesium

## Abstract

The choice of electrolyte has a crucial influence on the performance of rechargeable magnesium batteries. In multivalent electrolytes an agglomeration of ions to pairs or bigger clusters may affect the transport in the electrolyte and the reaction at the electrodes. In this work the formation of clusters is included in a general model for magnesium batteries. In this model, the effect of cluster formation on transport, thermodynamics and kinetics is consistently taken into account. The model is used to analyze the effect of ion clustering in magnesium tetrakis(hexafluoroisopropyloxy)borate in dimethoxyethane as electrolyte. It becomes apparent that ion agglomeration is able to explain experimentally observed phenomena at high salt concentrations.

## Graphical abstract

**Raised overpotential by clustering?**! A new continuum model that considers ion aggregation is presented and applied to magnesium tetrakis(hexafluoroisopropyloxy)borate in dimethoxyethane as electrolyte. Thereby, the properties of ion clusters and their effects on the battery performance are analyzed. Simulations imply that cluster formation can be essential to explain the concentration‐dependence of experimental data.

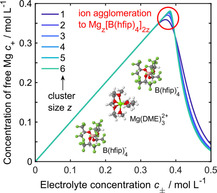

The main requirements for next‐generation batteries are a high energy density, a high safety, and a sufficient availability of raw materials at low cost. Compared to the state‐of‐the‐art Li‐ion battery technology, metal anodes are key to significantly higher specific capacities.^1^, ^2^, ^3^ Thereby, it is necessary to avoid capacity loss and short circuits caused by the growth of dendritic structures. In contrast to many other metals, magnesium prefers higher coordinated structures, which potentially enables a dendrite‐free and, therefore, safe cycling of the battery.^4^, ^5^ Another prominent advantage of magnesium is its natural abundance, which allows economic and sustainable large‐scale application of magnesium‐based battery technology.^3^, ^6^

The bivalency of the magnesium cations leads not only to a very high volumetric capacity but also to strong electrostatic interactions with the anion as well as with the solvent. Therefore, the solvation of the ions is always competing with their association, which can usually be seen in poor salt solubility and/or ionic conductivity. Indeed, it was found that ion pairs and bigger clusters are formed in many magnesium‐based electrolytes, for example, Mg(BH_4_)_2_, Mg(TFSI)_2_, or MgCl_2_.^7^, ^8^, ^9^, ^10^, ^11^, ^12^, ^13^, ^14^, ^15^ Thereby, the formation and size of the agglomerates strongly depends on the anion, the solvent, the concentration, and the electric field strength. Such clustering significantly affects electrolyte properties: clusters effectively screen the double charge of the magnesium cation, thus reducing charge density and interactions between the ionic agglomerates; but at the same time, the larger size of the clusters reduces their diffusivity, lowers ionic conductivity, and sterically hinders the charge transfer at the electrode.

As a first step towards understanding and minimizing the negative impact of ion clusters on battery performance we analyze the thermodynamics of the cluster formation equilibrium. For obtaining a predictive model, ion aggregation is consistently coupled to the transport of dissolved species in the electrolyte and the charge‐transfer reaction at the interface of a symmetric magnesium battery cell.

To date, experimental studies of ion pairs and clusters in magnesium‐based electrolytes, which are available in literature, are limited to the detection of the agglomerates. More detailed studies on the properties of prenucleation clusters have been done with aqueous calcium carbonate solutions for instance.^16^, ^17^, ^18^ Even though the physical properties of such a system differ significantly from the ones of magnesium‐based electrolytes with low‐dielectric‐constant solvents, it provides valuable information on the fundamental characteristics of prenucleation clusters. For instance, it was found that the clusters are neutral, thermodynamically stable species, which exist in under‐ and supersaturated solutions. Thereby, fast kinetics are observed, which leads to the conclusion that the activation barriers of the cluster equilibrium are negligible compared to the thermal energy. Moreover, ion aggregation is endothermic and, therefore, its driving force needs to be entropic in nature.

Since the release of solvent molecules from the solvation shells of the ions seems to be the driving factor for the cluster formation, we include the solvation in our description of the cluster formation equilibrium. In general, the ion aggregation in 2:1 magnesium electrolytes is given by

zMg(Sol)2+w+2zA(Sol)-x←→MgzA2z(Sol)zw+2zx-y+ySol

where A and Sol denote the anion and solvent, *w* and *x* are the solvation number of the magnesium cation and the corresponding anion, *y* is the number of released solvent molecules per cluster, and *z* describes the size of the neutral clusters, which consist out of 3*z* ions. This general cluster formation equilibrium can be described by the law of mass action [Eq. (1)], which correlates the equilibrium constant *K* with the activities *a* of the magnesium cation (+), the anion (−), the solvent (0), and the clusters (c).

(1) $$K=\frac{a_{c}a_{0}^{y}}{a_{+}^{z}a_{-}^{2z}}=\frac{c_{c}c_{0}^{y}}{\gamma_{+}^{z}c_{+}^{z}\gamma_{-}^{2z}c_{-}^{2z}}$$

For neutral species (cluster and solvent) it is assumed that the activity is well described by their concentration (*a*_0/c_ *≈c*_0/c_). For the dissolved ions we have to take non‐ideal behavior into account (*a*_+/−_=*γ*_+/−_⋅*c*_+/−_), because the concentration of the magnesium salt in the electrolyte is usually quite high and the high charge density of the bivalent magnesium cation leads to strong coulombic interactions. Since experimental data for the concentration‐dependent activity coefficients *γ* of magnesium electrolytes are very rare in literature, we use the modified Davis equation^19^ to describe the non‐ideality [Eqs. (S1)–(S4) in the Supporting Information].

The law of mass action [Eq. (1)] and the mass conservation in the equilibrium [Eqs. (S5)–(S7)] relate the concentrations of the four electrolyte species to the salt concentration *c*_±_ and can be coupled to our thermodynamically consistent transport theory [Eqs. (S14)–(S16)], which was presented in earlier work.^20^ The equation system is simplified for an isothermal process (*T*=298.15 K), adapted to the bivalency of magnesium *z*_+_=2, and solved for the magnesium salt concentration *c*_±_, the electrochemical potential of the electrolyte *ϕ*_e_, and the electric potential of the electrode *Φ*_s_.

The effect of the clusters on the activity of the magnesium ions is considered via the effective chemical *μ*, which can be described by Equation (2):

(2) $$\mu =\sum_{i}\frac{\partial c_{i}}{\partial c_{\pm }}\mu_{i}$$

where *i* denotes the existing species in the electrolyte, which in our case are the magnesium cation, the anion, the solvent, and the cluster (*i*=+, −, 0, c). The derivative of the effective chemical potential $\frac{\partial \mu }{\partial c{}_{\pm }}$ is an important part of the transport equations for the electrolyte [Eq. (S14)] and can be written as a function of the thermodynamic factor *f*_thermo_ [Eq. (3)]

(3) $$\frac{\partial \mu }{\partial c_{\pm }}=\frac{RT}{c_{\pm }}\cdot f_{\mathrm{thermo}}$$

Thereby, the thermodynamic factor, which describes all interactions between the species, is defined by Equation (4):

(4) $$f_{\mathrm{thermo}}=\sum_{i}\frac{\partial c_{i}}{\partial c_{\pm }}\cdot \frac{\partial ln\gamma_{i}}{\partial lnc_{\pm }}+{\left(\frac{\partial c_{i}}{\partial c_{\pm }}\right)}^{2}\cdot \frac{c_{\pm }}{c_{i}}$$

The activity coefficient of the neutral cluster *γ*_c_ and solvent *γ*_0_ are assumed to be 1, whereas the activity coefficients of the magnesium cation *γ*_+_ and the corresponding anion γ_−_ are given by the modified Davis equation [Eq. (S1)]. The correlation between the salt concentration *c*_±_ and the individual concentrations of the four electrolyte species *c_i_* results from the cluster formation equilibrium [Eqs. (1) and (S5)–(S7)]. The partial derivatives of the species concentrations and activity coefficients are determined numerically. A detailed derivation of the effective chemical potential *μ* and the thermodynamic factor *f*_thermo_ is given in the Supporting Information.

The electron transfer reaction at the interface is described by the Butler–Volmer approach. In principle, magnesium can be plated from the solvated cations as well as from the clusters. Since the magnesium cations need to get very close to the electrode surface for the electron‐transfer reaction and the clusters are large, it is assumed that only one magnesium cation per cluster can undergo the charge transfer at the interface. Moreover, the electrode surface is limited and, therefore, the two electroactive species will compete for reaction sites. Therefore, we have to consider steric effects, especially since the clusters are significantly larger than the solvated magnesium ions. This is done by introducing a weighting factor into the Butler–Volmer equation [Eq. (5)], which is based on the radius *r* and concentration *c* of the two solvated, electroactive species (*j*=+, c). Thus, the current density across the electrode–electrolyte interface *i*_se_ is given by Equation (5):

(5) $$i_{\mathrm{se}}=\sum_{j}\frac{c_{j}r_{j}}{\sum_{j{}^{'}}c_{j{}^{'}}r_{j{}^{'}}}\cdot i_{j}^{0}\left(\exp\left[\frac{\alpha_{j}^{A}z_{+}F}{RT}\eta_{s}\right]-exp\left[\frac{-\alpha_{j}^{C}z+F}{RT}\eta_{s}\right]\right)$$

αAj and αCj with αAj +αCj =1 are the anodic and cathodic symmetry factors. It is assumed that the activity of the free magnesium ions and the magnesium ions bound in the clusters is similar. Therefore, the overpotential *η*_s_ is equal for both electroactive species and can be written in terms of the electrochemical potential of the electrolyte *ϕ*_e_ [Eq. (6)]:

(6) $$\eta {}_{s}=\Phi {}_{s}-\varphi {}_{e}-U{}_{0}$$

where *U*_0_ denotes the open‐circuit potential and is zero for a symmetric magnesium cell. The exchange current density i0j is given by Equation (7):

(7) $$i_{j}^{0}=k_{j}\cdot z_{+}F\cdot a_{j}^{\alpha_{j}^{A}}c_{S}^{\alpha_{j}^{C}}$$

In our model we assume that clusters are less prone toward redox reaction at the electrode than solvated magnesium ions since the electrostatic interactions between magnesium cations and corresponding anions should be stronger compared to interactions between magnesium ions and solvent molecules. Moreover, bulky anions should hamper the bound magnesium ions to get close to the electrode surface. Therefore, the rate constant of the charge transfer reaction *k*_j_ will be significantly smaller for the clusters. In principle, it should depend on how many magnesium ions are located at the surface of the cluster. Therefore, the rate constant of the cluster *k*_c_ can be related to the one of the solvated magnesium *k*_+_ by the volume fraction of the enclosed magnesium via Equation (8).

(8) $$k_{c}=\frac{V_{+}}{V_{+}+2V_{-}}\cdot k_{+}=\frac{r{{}^{'}}_{+}^{3}}{r{{}^{'}}_{+}^{3}+2r{{}^{'}}_{-}^{3}}\cdot k_{+}$$

The radius of the cluster can be estimated by assuming that there is no solvent encased in the cluster. From the number and radius *r′* of the unsolvated ions, which form the cluster, follows Equation (9):

(9) $$r_{c}={\left(\frac{1}{\epsilon_{c}}\cdot z\cdot \left(r{{}^{'}}_{+}^{3}+2r{{}^{'}}_{-}^{3}\right)\right)}^{\frac{1}{3}}+r_{\mathrm{Sol}}$$

where *ϵ*_c_≤0.74 describes the packing density of the ions. The minimum value for the cluster radius is obtained when closest packing is assumed (*ϵ*_c_=0.74). Additionally, the solvation shell of the cluster (*r*_Sol_) has to be considered.

To test our newly developed continuum model, we apply it to the state‐of‐the‐art chloride‐free magnesium tetrakis(hexafluoroisopropyloxy)borate/dimethoxyethane (Mg[B(hfip)_4_]_2_/DME) electrolyte.^21^, ^22^ The set of equations is discretized by finite volumes and numerically solved for different electrolyte concentrations and current densities. The model parameters are either derived from experimental data or results of DFT calculations (Table 1). There is no concentration‐dependent experimental data of diffusion coefficients and transference numbers available in the literature. Therefore, the transference number is taken to be constant. In the case of the diffusion coefficient *D* the influence of the cluster can qualitatively be considered by the Stokes–Einstein equation, which describes an inverse dependence of the diffusion coefficient on the hydrodynamic radius. This leads to the following relation [Eq. (10)]:

(10) $$D_{c}=\frac{r_{+}}{r_{c}}\cdot D_{+}$$

**Table 1:** Parameters for the simulation of a symmetric Mg cell with Mg[B(hfip)4]2/DME electrolyte.

Parameter	Value	Source
*Transport*		
*κ* ${}_{c{}_{\pm }}$	0–1.1 S m^−1^	22
*D*_+_	1×10^−10^ m^2^ s^−1^	–
*t*_+_	0.21	experiment^[a]^
*Kinetics*		
*k*_+_	1.3×10^−9^ m s^−1^	23
αA+ =αC+	0.5	–
αAc =αCc	0.5	–
Cluster		
*w*	3	21, DFT^[a]^
*x*	0	DFT^[a]^
*y*	3*z*	24, DFT^[a]^
*r*_+_	487 pm	DFT^[a]^
*r*_−_=r'-	584 pm	DFT^[a]^
r'+	80 pm	25
*ϵ*_c_	0.74	
*Solvent*		
*ρ*_(DME)_^[b]^	868 g m^−3^	26
*ϵ*_*r*(DME)_^[c]^	7.3	26

[a] The technical details on the DFT calculations and the measurement of the transference number can be found in the Supporting Information. [b] The concentration of DME *c*_0_ is calculated from its molar mass and its density *ρ*. [c] *ϵ_r_* denotes the relative permittivity.

The effective diffusion coefficient, which is needed for the transport equation, is given by the weighted, harmonic mean [Eq. (11)]:

(11) $$D=\frac{c_{+}+c_{c}}{\frac{c_{+}}{D_{+}}+\frac{c_{c}}{D_{c}}}$$

In principle, a higher electrolyte concentration enables faster electron transfer kinetics [Eqs. (5) and (7)]. Furthermore, the experimental data for the ionic conductivity^22^ implies that there is no significant transport limitation in the salt concentration range between 0.2–0.5 m. Therefore, a decrease of the overpotential with increasing electrolyte concentration is expected when no ion aggregation takes place (Figure 1). Since experimental studies report increasing overpotentials with increasing electrolyte concentration^22^, ^27^ there has to be an additional process that has a negative impact on the ion mobility in the electrolyte and/or on the electrochemical reaction at the electrodes. Our simulations including the effect of cluster formation indicate that this discrepancy can indeed be explained by ion agglomeration (Figure 1).

**Figure 1:**
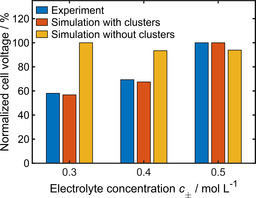
Overpotential of Mg plating in symmetric Mg cells at 1 mA cm^−2^: Qualitative comparison of experimental data^22^, ^27^ and results from simulations with (*K=*1, *z=*3) and without considering cluster formation.

Since the exact properties of the Mg_*z*_[B(hfip)_4_]_2z_ ion clusters are not known yet, parameter studies were performed to investigate the influence of the stability (*K*) and size (*z*) of the agglomerates on the cluster‐formation equilibrium. Equilibrium constants *K* from 10^−5^ to 10^5^ and cluster sizes *z* from 1 to 10 were analyzed (Figures 2 and S3). It is known that in low dielectric solvents contact and solvent‐separated ion pairs are present at very low salt concentrations.^24^ The simple evaluation of the cluster‐formation equilibrium (Figure 2) is not able to describe this behavior. Since this work focuses on higher electrolyte concentrations, as they are used in experiments, ion pairing at dilute concentrations is not considered in the model. The analysis of the cluster formation equilibrium (Figure 2) shows that the amount of free magnesium ions *c*_+_ increases linearly with the electrolyte concentration *c*_±_ until a critical concentration is reached, where cluster formation occurs. Subsequently, the concentration of free magnesium ions quickly drops before it slowly approaches zero and almost all magnesium ions are trapped in ionic agglomerates. It can be seen that the impact of the cluster size is quite small (Figure 2 b), whereas the cluster stability more significantly affects the critical electrolyte concentration for ion agglomeration (Figure 2 a). Moreover, it becomes obvious, that the electrolyte concentrations, which were used in the experiments (0.3–0.5 m),^22^, ^27^ are quite close to the critical concentration, no matter how big and stable the clusters are (Figures 1 and Figure 2). Furthermore, the evaluation of the cluster‐formation equilibrium (Figure S3) of the 0.4 m electrolyte shows that the formation of medium‐to‐big sized clusters is thermodynamically favorable when the clusters are unstable (*K*<1). With increasing equilibrium constant the formation of smaller clusters becomes advantageous.

**Figure 2:**
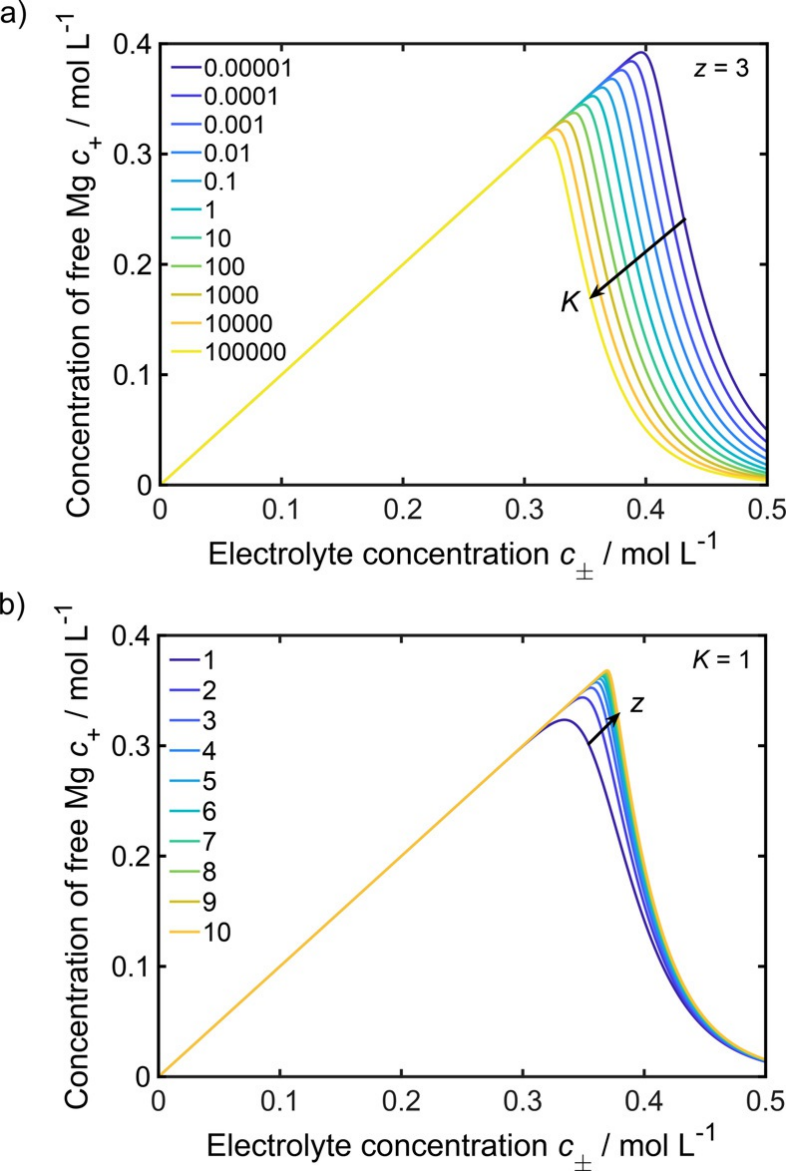
Influence of equilibrium constant *K* (a) and cluster size *z* (b) on ion aggregation.

In a next step the impact of cluster size and stability on the overpotential of a symmetric magnesium cell is evaluated. This is done for nine different electrolyte concentrations between 0.1 and 0.5 m as well as for two different current densities (0.1 and 1 mA cm^−2^). The corresponding concentrations of free Mg^2+^ for the analyzed *K* and *z* range can be found in the Supporting Information (Figure S4). For small electrolyte concentrations (*c*_±_<0.2 m) no significant cluster formation could be observed, independent of cluster size and stability.

Figure 3 shows simulation results for the 0.4 m electrolyte at 0.1 mA cm^−2^. In general, stable clusters lead to a higher overpotential. This expected behavior becomes more pronounced the smaller the clusters are and the closer the electrolyte concentration is to the critical concentration (Figure S5). Moreover, it can be seen that when the clusters are thermodynamically unstable (*K*<1) larger clusters affect the battery performance stronger than smaller ones. The opposite behavior is observed for stable (*K*>1) ion clusters.

**Figure 3:**
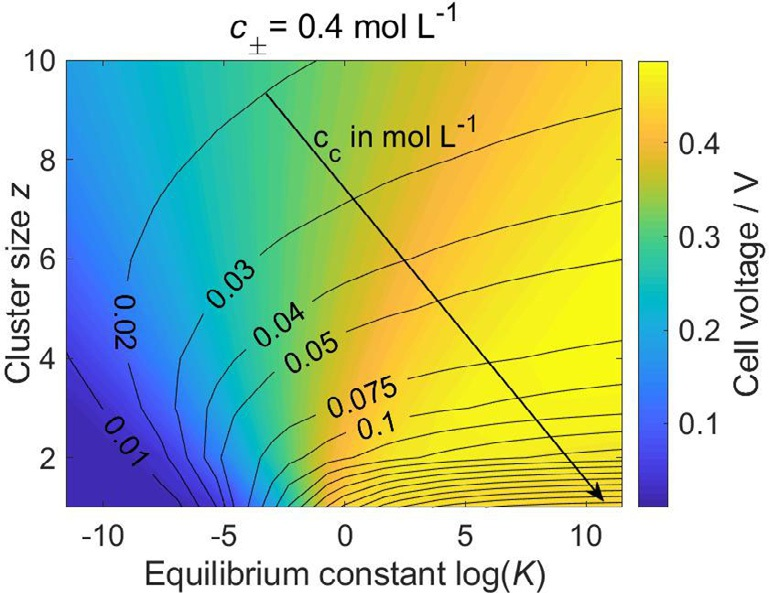
Influence of *K* and *z* on the overpotential and the mean cluster concentration *c*_c_ in symmetric Mg cells with 0.4 m Mg[B(hfip)_4_]_2_/DME electrolyte at a current density of 0.1 mA cm^−2^.

Comparison of Figures 3 and S3 implies, that there is an inverse correlation between the concentration of free Mg^2+^ (*c*_+_) and the overpotential. The exact relation between the cell voltage and the amount of free magnesium ions for the analyzed electrolyte concentrations is shown in Figure 4. As expected, an inverse linear behavior can be seen over a wide range, especially at low current densities. Consequently, the main effect of cluster formation seems to be that the concentration of the main electrochemical active species Mg^2+^ (*c*_+_) is reduced. However, there are also significant deviations from linearity (Figure 4), which reflect the impact of the clusters on ion mobility in the electrolyte and the reaction at the electrodes taken into account in our model. By including and excluding the individual effects in the simulations (Figure S7) and by analyzing the kinetics of the electron‐transfer reaction (Figure S6) as well as the concentration gradients (Figure S9), the observed behavior of the overpotential (Figure 4) can be explained. A detailed discussion of the contribution of individual processes can be found in the Supporting Information.

**Figure 4:**
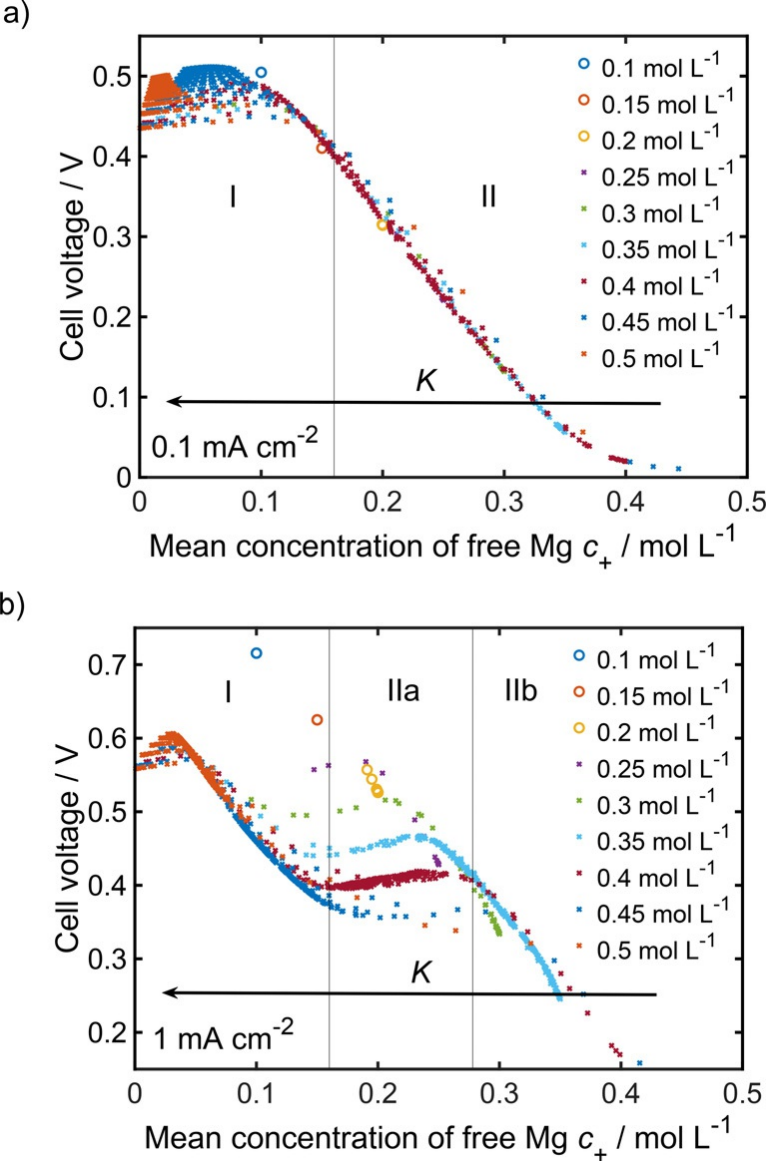
Parameter study: Correlation between the overpotential and the mean concentration of free magnesium ions in symmetric Mg cells at current densities of 0.1 mA cm^−2^ (a) and 1 mA cm^−2^ (b) with varying initial salt concentration.

The variation of the overpotential at similar *c*_+_ in Figure 4 is caused by the influence of the cluster size *z* on the kinetics of the electron transfer reaction. The steric hindrance dominates at higher *c*_+_ (lower *c*_c_, Figure 4 a, region II), which means that bigger clusters are slightly advantageous for the overpotential (Figure S6). The pronounced fluctuations of the overpotential at low *c*_+_ (Figure 4 a, region I) can be assigned to the electrochemical reactivity of the clusters, which provides a parallel route for magnesium plating and stripping. Therefore, the cluster size has a significant influence on the Butler–Volmer reaction rate and consequently on the overpotential. In contrast to the region II, which is dominated by steric effects, smaller clusters are favorable at low *c*_+_.

The maximum of the overpotential at very low *c*_+_ (Figure 4 a, region I) is mainly caused by the activity of the free magnesium ions (Figure S6), which is considered in the Butler–Volmer reaction rate [Eq. (7)]. Because of the coulombic interactions with the anion the activity of the magnesium ions first tends to decrease, which causes an increase of the overpotential. After a critical value is reached the activity starts to increase with the concentration, which enhances the kinetics of the charge transfer and reduces the overpotential. Another contribution to the maximum of the overpotential at low *c*_+_ can be assigned to the electrochemical reactivity of the clusters. In the region of small *c*_+_ the cluster concentration *c*_c_ is very high and the additional plating from the clusters overcompensates the steric hindrance of the plating from free magnesium ions, which leads to an enhancement of the kinetics.

Figure 4 b shows the same graph at a higher current density. In our simulations we observe a pronounced shoulder, where the overpotential remains constant over a wide range of *c*_*+*_ (Figure 4 b, region IIa). This feature can be assigned to a transport limitation in the electrolyte, which leads to high concentration gradients and causes a locally smaller or higher electrolyte concentration *c*_±_ at the two electrodes, respectively. Consequently, the cluster formation will be diminished or enhanced, which directly affects the kinetics of the electron transfer reaction. Therefore, the behavior of the cell voltage at high *c*_+_ (region IIb) is determined by stripping (Figure S8) whereas plating is responsible for the trends of the overpotential in the low *c*_+_ range (region I and IIa).

From experiments it is known that the overpotential for the 0.3 m and 0.4 m Mg[B(hfip)_4_]_2_/DME electrolyte is quite similar at a current density of 0.1 mA cm^−2^.^22^ This information is used to get more insight about the values for *K* and *z* (Figure S10 a). It was found that the value for the equilibrium constant *K* decreases exponentially with the cluster size *z*. Consequently, bigger clusters need to be less stable than smaller ones to have the same effect on the overpotential. Interestingly, the ion clusters in the Mg[B(hfip)_4_]_2_/DME electrolyte seem to be thermodynamically unstable (*K*<1), which is in contrast to CaCO_3_ prenucleation clusters that were found in aqueous solution^16^, ^17^, ^18^ as well as to Mg(TFSI)^+^ ion pairs in DME and diglyme.^15^ As expected, the adverse impact of ion clusters is more pronounced the bigger the clusters are. However, the influence of the cluster size is very small (Figure S10 b). Therefore, simulations with clusters of the size *z=*1 were used to identify the electrolyte concentration with the best performance in terms of a small overpotential (Figure 5). It is found that a salt concentration between 0.3 m and 0.4 m is ideal, whereby the electrolyte concentration showing the lowest overpotential becomes slightly lower with increasing current density. Moreover, there is a correlation between the ideal electrolyte concentration and the mean cluster concentration in the electrolyte. The adverse effects of the clusters become relevant for the overpotential almost as soon as they are formed (*c*_c_≈0.01 mol L^−1^). Furthermore, it is found, that the overpotential of cells with electrolyte concentrations of around 0.45 m decreases at high current densities (Figures 5 and S10 b). This behavior is counter intuitive and certainly requires more investigation.

**Figure 5:**
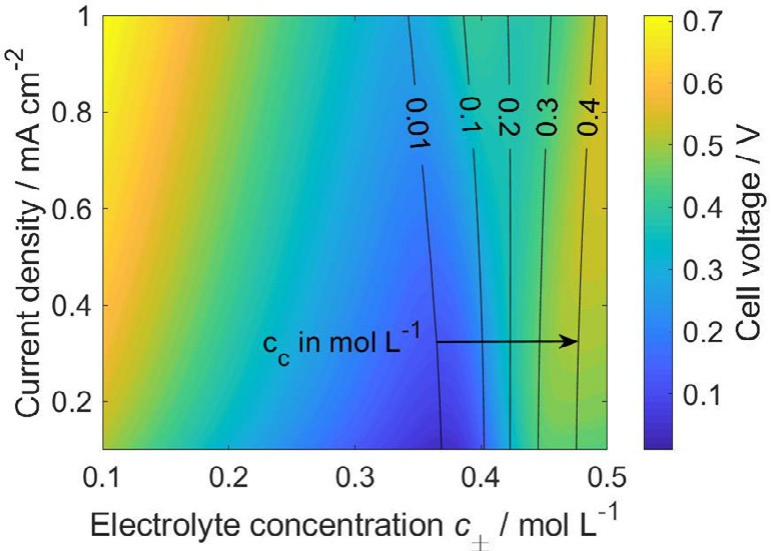
Influence of the electrolyte concentration *c*_*±*_ and the current density on the overpotential and *c*_c_ for clusters with the size *z=*1 and the equilibrium constant ln(*K*)=−3.97.

In summary, we consistently included the aggregation of ions to clusters in our model for electrolytes containing magnesium salts. This general model was applied to a symmetric magnesium cell with magnesium tetrakis(hexafluoroisopropyloxy)borate/dimethoxyethane (Mg[B(hfip)_4_]_2_/DME) as electrolyte to develop a better understanding of the influence of the cluster formation on the overall battery performance. Parameter studies were done to analyze the impact of the electrolyte concentration and the current density as well as the stability and size of ion clusters. In general, there is a critical electrolyte concentration at which the amount of clusters is high enough (>0.01 m) to have a negative impact on battery performance. Therefore, the clusters mainly affect the kinetics of the charge‐transfer reaction. Additionally, the clusters reduce the ion mobility in the electrolyte, but transport limitations were only found at high current densities. Surprisingly, for certain cluster properties and electrolyte concentrations the transport limitations may even be advantageous for battery performance, but in this case the overpotential is significantly higher than the predicted minimum. All in all, cluster formation is key to reproduce the qualitative trends in the experimental data at different electrolyte concentrations. Finally, our simulations predict that at current densities between 0.1 and 1 mA cm^−2^ the best battery performance can be found at electrolyte concentrations around 0.35 m. Most of the analysis in this work focuses on the performance of symmetric magnesium cells, which are of limited practical relevance. However, as demonstrated in Figure S8, similar analysis can also be performed for cells including reference electrodes or full cell setups. Therefore, the model presented in this article provides basis for the theoretical analysis and optimization of magnesium electrolytes showing ion clustering. Further research should include the transfer of the model to other magnesium electrolytes such as Mg(TFSI)_2_ and especially chloride‐containing systems. Moreover, detailed models for the analysis of the electrode–electrolyte interface, for example, including degradation effects,^28^ need to be developed.

## Conflict of interest

*The authors declare no conflict of interest*.

## Supporting information

As a service to our authors and readers, this journal provides supporting information supplied by the authors. Such materials are peer reviewed and may be re‐organized for online delivery, but are not copy‐edited or typeset. Technical support issues arising from supporting information (other than missing files) should be addressed to the authors.

Supplementary
