# Supplementary material for: Modeling of Ion Agglomeration in Magnesium Electrolytes and its Impacts on Battery Performance
Source: ChemSusChem. 2020 Jun 29;13(14):3599–604. doi: 10.1002/cssc.202001034 (PMC7496337; doi:10.1002/cssc.202001034)
Supplement: Supplementary file 1 — Supplementary [file CSSC-13-3599-s001.pdf]

ChemSusChem

Supporting Information

Modeling of Ion Agglomeration in Magnesium Electrolytes and its Impacts on Battery Performance

Janina Drews,*[a, b] Timo Danner,[a, b] Piotr Jankowski,[c] Tejs Vegge,[c]
Juan Maria García Lastra,[c] Runyu Liu,[d] Zhirong Zhao-Karger,[d] Maximilian Fichtner,[b, d] and
Arnulf Latz[a, b, e]

1 Details of the model

1.1 Modified Davis equation [1]

$$\ln \gamma_i = -\frac{A z_i^2 \sqrt{I}}{1 + r_i B \sqrt{I}} + \frac{C_{(I)} A z_i^2 \sqrt{I}}{\sqrt{1000}} \tag{S1}$$

$$A = \frac{\sqrt{2} F^2 e_0}{8\pi (\varepsilon_r \varepsilon_0 R T)^{3/2}} \tag{S2}$$

$$B = \sqrt{\frac{2F^2}{\varepsilon_r \varepsilon_0 R T}} \tag{S3}$$

$$C_{(I)} = -3,33 \cdot 10^{-5} \cdot I + 0,2 \tag{S4}$$

The modified Davis equation is an extention of the Debye-Hückel model, which enables the determination of activity coefficients of concentrated electrolytes with ionic strengths $I = \frac{1}{2} \cdot \sum_i z_i^2 c_i$ up to $1500\,\frac{\text{mmol}}{\text{L}}$ [1]. For the ion i only its charge number z_i, its hydrodynamic radius r_i, the dielectric constant of the solvent ε_r and the temperature T are needed in addition to the fundamental physical constants, which are the Faraday constant F, the elementary charge e_0, the vacuum permittivity ε_0 and the ideal gas constant R.

This modified Davis equation certainly is a crude approximation of ion activity and additional measurements will improve the quality of predictions. However, we expect that the model is able to describe qualitative trends in concentrated magnesium electrolytes.

1.2 Conservation of mass in the cluster formation equilibrium

$$\mathrm{z \cdot Mg(Sol)_w}^{2+} + 2\,\mathrm{z \cdot A(Sol)_x}^{-} \rightleftharpoons \mathrm{Mg_z A_{2z} (Sol)_{zw+2zx\text{-}y}} + \mathrm{y \cdot Sol},$$

The concentrations of the four species of the cluster formation equilibrium and the concentration of the magnesium salt MgA_2 $c_\pm$ are related by the conservation of mass. The solvent concentration is additionally determined by its density ρ_{Sol} and its molar mass M_{Sol}. The total concentrations c^0 of magnesium, anion and solvent can be described by

$$c^0_{Mg} = c_+ + z \cdot c_c = c_\pm \tag{S5}$$

$$c^0_A = c_- + 2z \cdot c_c = 2c_\pm \tag{S6}$$

$$c^0_{Sol} = w \cdot c_+ + x \cdot c_- + (zw + 2zx - y) \cdot c_c + c_0 = \frac{\rho_{Sol}}{M_{Sol}} \tag{S7}$$

Equation (S5) and (S6) represent the stoichiometry of the magnesium salt, which is given by

$$c_- = 2c_+. \tag{S8}$$

1.3 Transport theory [2]

1.3.1 Effective chemical potential and thermodynamic factor

The effective chemical potential μ is used to simplify the fundamental thermodynamic relation for a polarizable system in an external electromagnetic field [2]

$$\partial e = T\partial s + \mu_+ \partial c_+ + \mu_- \partial c_- + \mu_0 \partial c_0 + \mu_c \partial c_c + \vec{E}\cdot\partial\vec{D} + \vec{H}\cdot\partial\vec{B} \tag{S9}$$

Thereby, the sum $\sum_i \mu_i \partial c_i$ $(i = +,-,0,c)$ is required, which can be described in terms of the salt concentration $c_\pm$.

$$\sum_i \mu_i \partial c_i = \sum_i \frac{\partial c_i}{\partial c_\pm}\mu_i \cdot \partial c_\pm = \mu \cdot \partial c_\pm \tag{S10}$$

Consequently, the effective chemical potential is given by Equation (2). For the transport equations [Eq. (S14)] the derivative of the effective chemical potential is needed.

$$\frac{\partial \mu}{\partial c_\pm} = \sum_i \frac{\partial c_i}{\partial c_\pm}\frac{\partial \mu_i}{\partial c_\pm} \tag{S11}$$

Thereby, the derivative of the individual chemical potentials μ_i with respect to the salt concentration $c_\pm$ can be described by using the definition of the chemical potential $\mu_i = \mu_i^0 + RT\ln a_i$ as well as the definition of the activity $a_i = \gamma_i c_i$:

$$\begin{aligned}\frac{\partial \mu_i}{\partial c_\pm} &= \frac{\partial c_i}{\partial c_\pm}\frac{\partial \mu_i}{\partial c_i} = RT\cdot\frac{\partial c_i}{\partial c_\pm}\cdot\left(\frac{\partial \ln\gamma_i}{\partial c_i} + \frac{\partial \ln c_i}{\partial c_i}\right) = \frac{RT}{c_\pm}\cdot\frac{\partial c_i}{\partial c_\pm}\left(c_\pm\cdot\frac{\partial \ln\gamma_i}{\partial c_i} + \frac{c_\pm}{c_i}\right)\\ &= \frac{RT}{c_\pm}\cdot\left(\frac{\partial \ln\gamma_i}{\partial \ln c_\pm} + \frac{\partial c_i}{\partial c_\pm}\cdot\frac{c_\pm}{c_i}\right)\end{aligned} \tag{S12}$$

Equation (S11) and (S12) lead to following relation:

$$\frac{\partial \mu}{\partial c_\pm} = \frac{RT}{c_\pm}\cdot\sum_i \frac{\partial c_i}{\partial c_\pm}\cdot\frac{\partial \ln\gamma_i}{\partial \ln c_\pm} + \left(\frac{\partial c_i}{\partial c_\pm}\right)^2\cdot\frac{c_\pm}{c_i} \tag{S13}$$

This final expression is described by Equation (3) and (4). Thereby, the derivatives of the individual concentrations c_i and activity coefficients γ_i of the species with respect to the salt concentration $c_\pm$ are determined numerically by using the cluster formation equilibrium [Eq. (1)] and S5-S7) and the Davis equation [Eq. (S1)-(S4)], respectively.

1.3.2 Transport equations

In the electrolyte charge conservation leads to the transport equation for the ionic current $\vec{j}_e$:

$$0 = -\vec{\nabla}\vec{j}_e = \vec{\nabla}(\kappa\vec{\nabla}\varphi_e) + \vec{\nabla}\left(\kappa\frac{t_+ - 1}{z_+F}\frac{\partial\mu}{\partial c_\pm}\vec{\nabla}c_\pm\right), \tag{S14}$$

where κ denotes the ionic conductivity of the electrolyte, t_+ the transference number of the magnesium cations and μ the effective chemical potential. Mass conservation results in following differential equation for the particle transport $\vec{N}_e$ in the electrolyte:

$$\frac{\partial c_\pm}{\partial t} = -\vec{\nabla}\vec{N}_e = \vec{\nabla}(D\vec{\nabla}c_\pm) - \vec{\nabla}\left(\frac{t_+}{z_+F}\vec{j}_e\right) \tag{S15}$$

where D is the interdiffusion coefficient. Important to note is, that when the transport coefficients of the electrolyte (κ, D and t_+) are experimentally determined in dependence of the salt concentration, the effect of the clusters on these parameters is already included and has not to be considered separately in our model. Since magnesium is a conversion electrode, there is no transport of ions in the solid phase ($\vec{N}_s = 0$) and the electric current $\vec{j}_s$ simplifies to

$$0 = -\vec{\nabla}\vec{j}_s = \vec{\nabla}(\sigma\vec{\nabla}\Phi_s). \tag{S16}$$

where σ stands for the electronic conductivity of the magnesium electrode.

2 Details of the parameterization

2.1 DFT calculations

All DFT calculations have been performed using 6-311++G(d,p) basis set and M06-2X functional as implemented in Gaussian16 B.01. Geometries of the species were optimized, and the local minima confirmed by frequency calculations. Solvation of the Mg^{2+} and $B(hfip)_4{}^-$ ions by DME molecules was studied by step-by-step introduction of solvent molecules around the ions (Figure S1). The stable 1st solvation shell of the Mg^{2+} cation was found to contain 3 DME molecules, and further introduced solvent molecules to the 2nd solvation shell were bonded significantly weaker (w=3). For $B(hfip)_4{}^-$ anion, interaction with solvent molecules was found to be much weaker even in the 1st solvation shell, thus can be neglected (x=0). Bonding energies of subsequent DME molecules, based on the electronic energies, as shown in Table S1. Size of the molecules was determined, by the radius of the sphere bounding all atoms with Van der Waals radii.

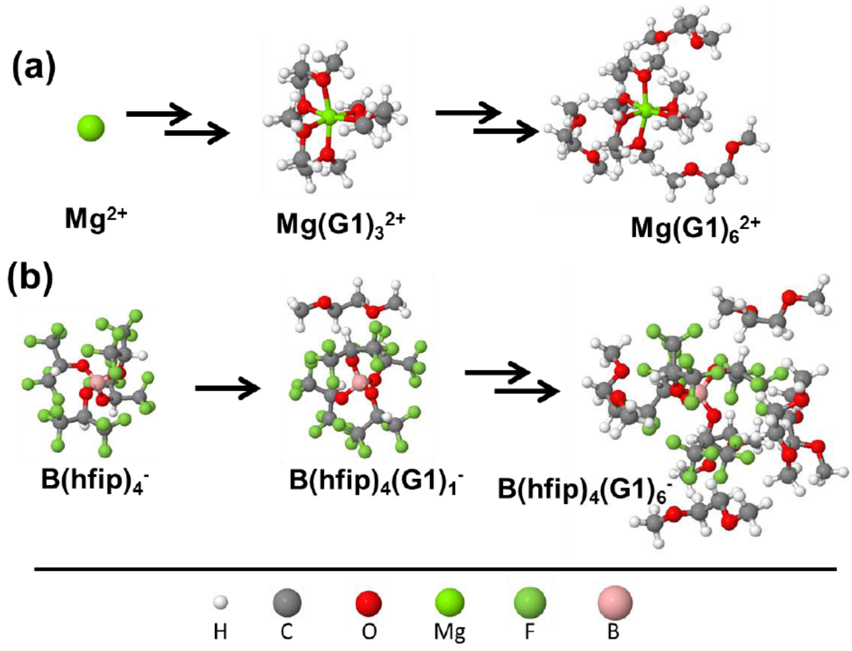

Figure S1 Optimized geometries of the solvation shell of Mg^{2+} (a) and $B(hfip)_4{}^-$ (b) ions.

Table S1 Binding energy of subsequent DME molecules in the solvation shell of Mg^{2+} and $B(hfip)_4{}^-$ ions. All values in $kJ\,mol^{-1}$.

Ion	1st	2nd	3rd	4th	5th	6th
Mg^{2+}	708	490	284	83	80	78
$B(hfip)_4{}^-$	55	35	35	34	30	23

2.2 Measurement of the transference number

0.3 M $Mg[B(bfip)_4]_2$ / DME electrolyte solution was prepared following the procedures as reported.[3] The electrochemical measurements were carried out with a Biologic VMP-3 potentiostat. Symmetric Mg|Mg swagelok cells were assembled with polished Mg discs (99.9%) and a glass fiber separator soaked with the electrolyte solution in a glovebox under argon atmosphere. The transference number t_+ was determined with the Bruce and Vincent formula:[4]

$$t_+ = \frac{I_{ss}(\Delta V - I_0 R_0)}{I_0(\Delta V - I_{ss} R_{ss})} \tag{S17}$$

where I_0 and I_{ss} are the initial and steady state currents, ΔV is the applied potential, t_+ is the cation transference number, R_0 and R_{ss} are the electrode resistance before and after polarization, respectively. The measurements were performed using potentialstatic method by applying a potential of 10 mV for 1 h (Figure S2a); impedances were measured in the frequency range of 0.05 – 100,000 Hz (Figure S2b). The experiments showed a transference number t_+ of 0.211 ± 0.003.

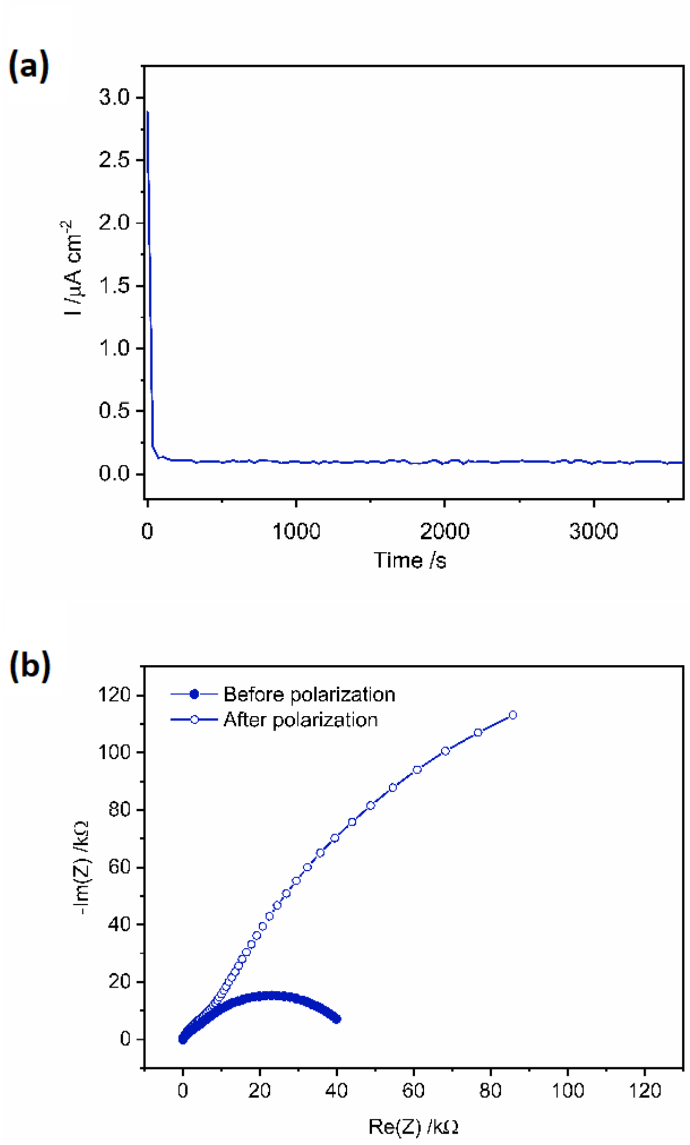

Figure S2 (a) Chronoamperogram of 0.3 M $Mg[B(bfip)_4]_2$ / DME with an applied voltage of 10 mV. (b) Nyquist plots before and after the polarization.

3 Application of the model to the $Mg[B(hfip)_4]_2$ / DME electrolyte

3.1 Influence of the cluster size and stability on the ion aggregation

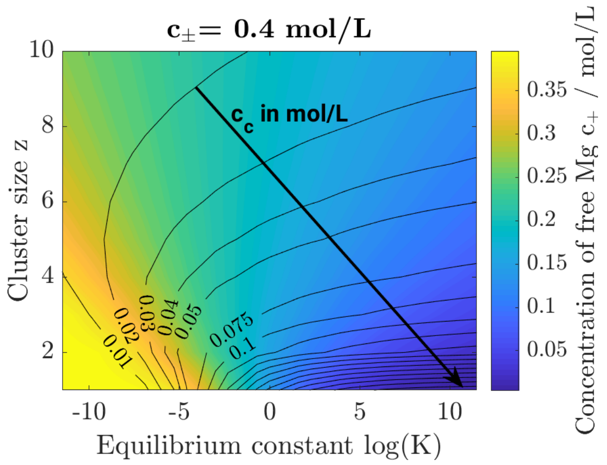

Figure S3 Influence of the equilibrium constant K and the cluster size z on the ion aggregation in a 0.4 M $Mg[B(hfip)_4]_2$ / DME electrolyte.

3.2 Influence of the cluster size and stability on the overpotential

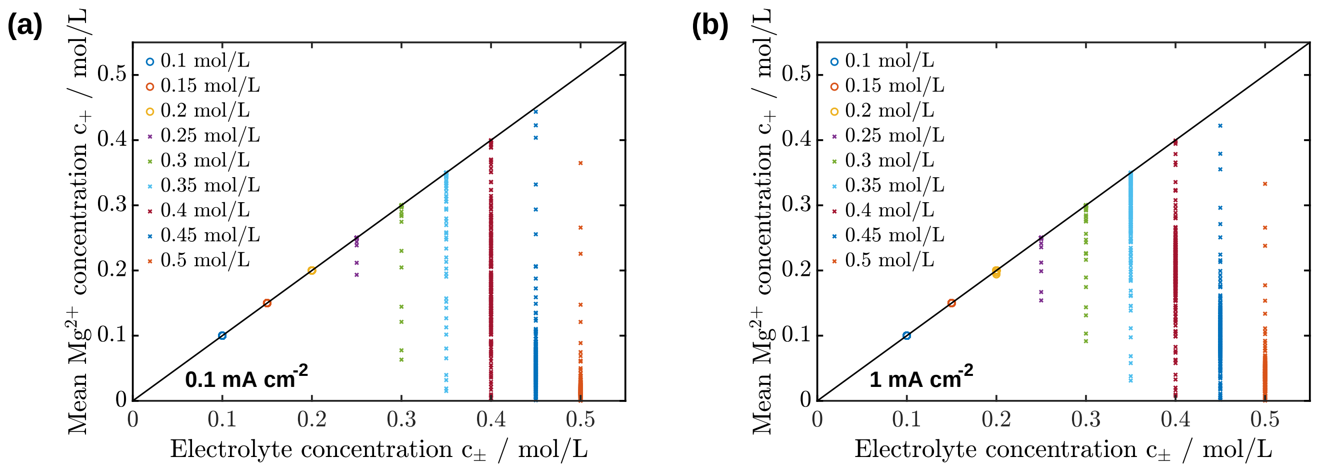

Figure S4 Overview over the concentration of free Mg^{2+} for the analyzed data points of the parameter study: $K = 10^{-5} - 10^{5}$, $z = 1 - 10$, $c_{\pm} = 0.1 - 0.5$ M and current densities of 0.1 mA cm^{-2} (a) and 1 mA cm^{-2} (b).

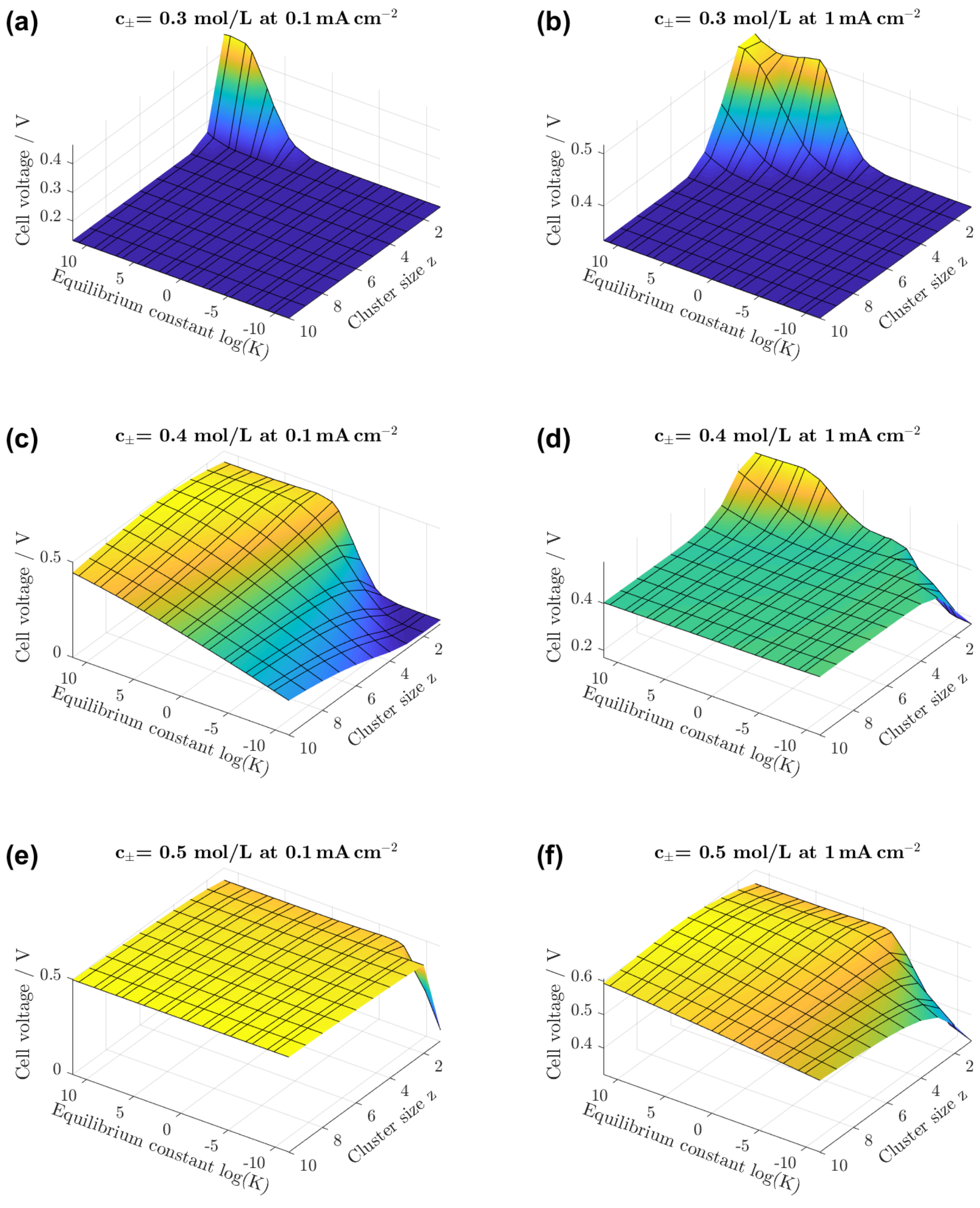

Figure S5 Influence of the equilibrium constant K and the cluster size z on the overpotential in symmetric Mg cells with 0.3 M (top) 0.4 M (middle) and 0.5 M (bottom) $Mg[B(hfip)_4]_2$ / DME electrolyte at current densities of 0.1 mA cm^{-2} (left) and 1 mA cm^{-2} (right).

The most significant influence of the equilibrium constant and the cluster size on the concentration of the ion clusters (Figure 2) and therefore on the overpotential (Figure S5) is observed for the 0.4 M electrolyte. Thereby the impact of K and z at high current densities is less pronounced. This is caused by the steeper concentration gradient in the electrochemical cell. During operation the electrolyte concentrations become significantly larger or smaller than 0.4 M at the the electrode surfaces and therefore aren't located in the region, where c_+ (and c_c) changes the most with $c_\pm$ anymore (Figure 2). This is in contrast to the 0.3 and 0.5 M electrolyte, where a higher current density causes a more significant impact of K and z, since the local electrolyte concentrations at the electrodes get closer to the critical concentration.

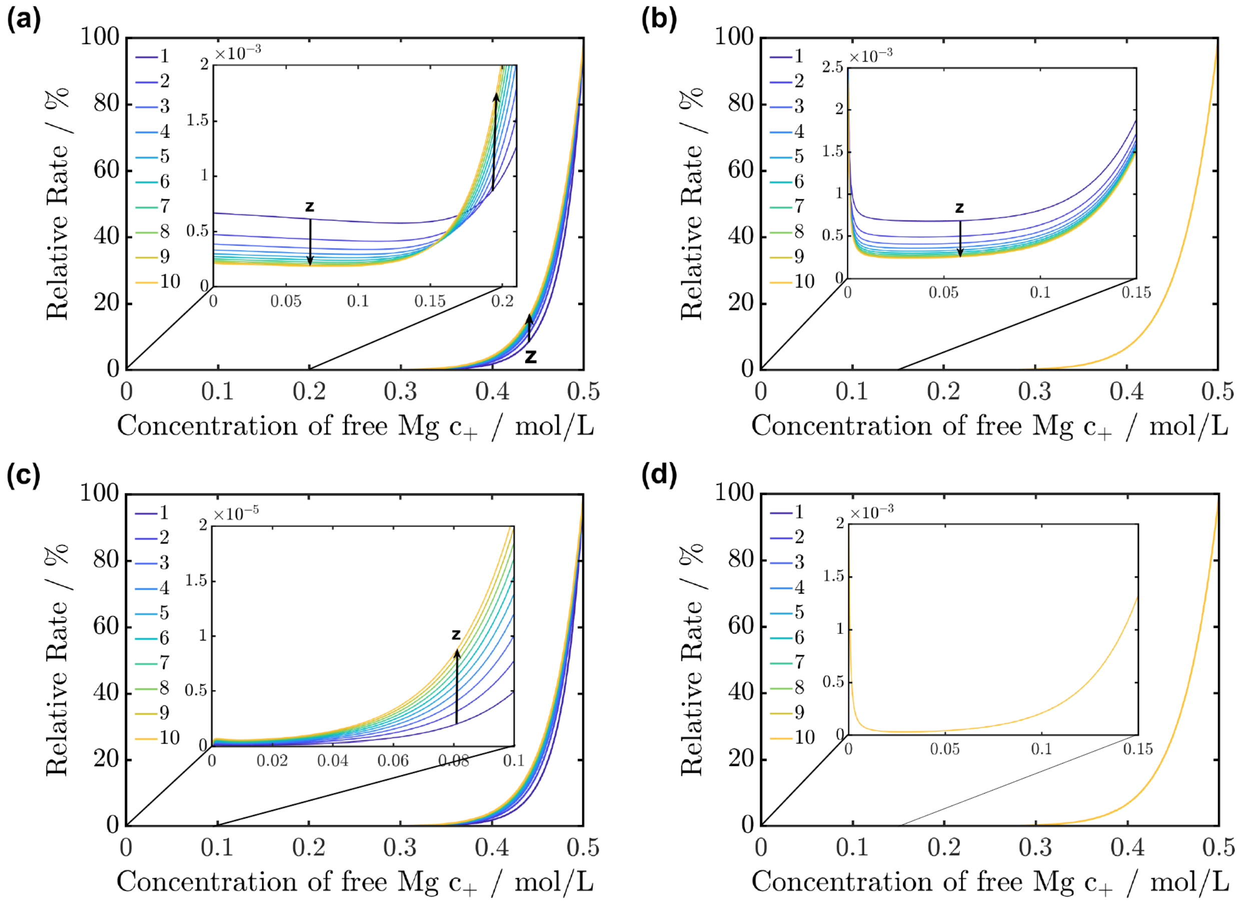

Figure S6 Analysis of different effects including the cluster size z on the relative Butler-Volmer rate ($c_{\pm} = 0.5\,\mathrm{M}$, $\eta_s = 1\,\mathrm{V}$). (a) all effects (b) without steric hindrance (c) inactive clusters (d) inactive clusters without steric hindrance.

Considering the kinetics of the electron transfer reaction at the electrode, our model includes two opposite effects: On the one hand the clusters sterically hinder the free magnesium in accessing the electrode surface, which implies, that a high cluster concentration is kinetically unfavorable. On the other hand it is possible to plate magnesium from the clusters and therefore a high cluster concentration might enhance the reaction rate. Figure S6 shows the impact of different sized clusters on the reaction rate for a 0.5 M $Mg[B(hfip)_4]_2$ / DME electrolyte. When neither the steric effects nor the electrochemical reactivity of the ion clusters is considered (Figure S6d) the Butler-Volmer rate is proportional to the magnesium activity. By considering only the steric effects (Figure S6c) it can be seen, that bigger clusters lead to a higher reaction rate than smaller ones. The steric hindrance is considered by the concentration and the radius of the agglomerates [Eq. (5)]. Since the cluster concentration c_c is proportional to $\frac{1}{z}$ [Eq. (S5)] and the radius of the cluster r_c only is proportional to $z^{\frac{1}{3}}$ [Eq. (9)] a higher concentration dominates over a smaller radius of the clusters. Therefore, at a similar concentration of free magnesium (c_+) the steric hindrance is higher for smaller clusters, which leads to an increase of the reaction rate with z. The opposite influence of the cluster size can be observed when only the reactivity of the clusters is considered (Figure. S6b). Since the rate constant for the magnesium plating from the clusters is mainly determined by the cluster concentration, the kinetics becomes faster for smaller clusters. By considering both effects of the clusters (Figure. S6a) it can be seen, that the influence of the steric hindrance dominates at higher c_+ and the influence of the additional plating from the clusters dominates at small c_+.

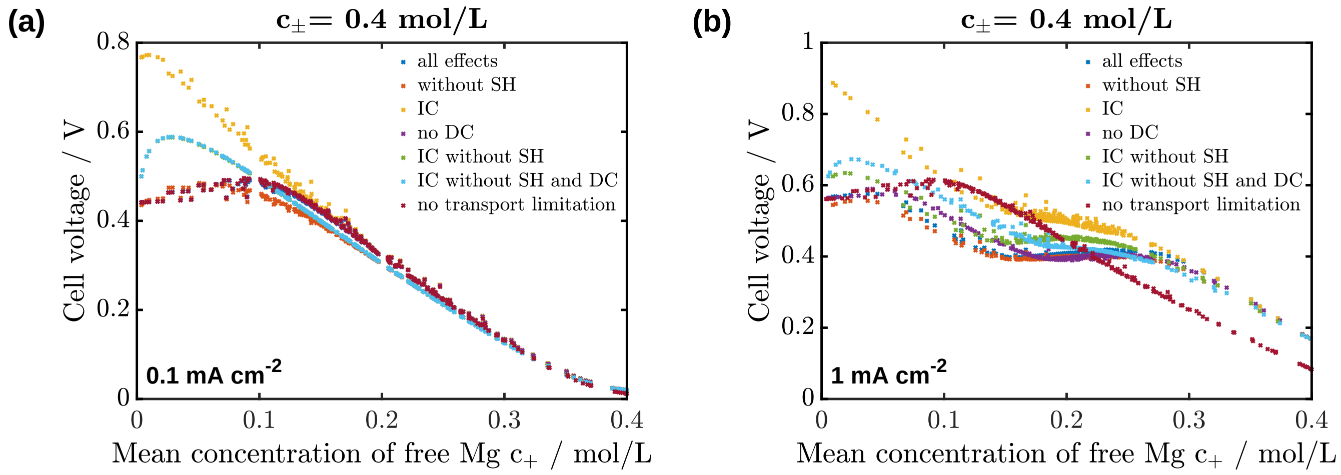

Figure S7 Parameter study: Analysis of the different effects of the clusters on the overpotential of a symmetric Mg cell with 0.4 M $Mg[B(hfip)_4]_2$ / DME electrolyte at current densities of 0.1 mA cm^{-2} (a)*and 1 mA cm^{-2} (b).
Abbreviations in the legend: SH = steric hindrance, IC = inactive clusters and DC = diffusion correction.

The different effects of the clusters on the overpotential are analyzed by including and excluding the impacts of the clusters on the kinetics of the charge transfer reaction (steric hindrance and reactivity of the clusters), as well as on the ion mobility (diffusion coefficient) in the simulations. The results are shown in Figure S7.

When no plating from the clusters is possible, the overpotential decreases almost linearly with c_+. As expected the inactivity of the clusters leads to a higher overpotential and is more pronounced at high c_c (low c_+). At the high current density this effect is already observable for quite high c_+. For the 0.4 M electrolyte the influence of the inactive clusters is significant for $c_+ \lesssim 0.3$ M at the high current density and for $c_+ \lesssim 0.15$ M at the low current density for instance (Figure S7).

Without considering the steric effects of the clusters on the reaction rate the behavior of the overpotential doesn't change as dramatically as when the clusters are assumed to be electrochemically inactive. If the clusters don't sterically hinder the plating from the free magnesium, the kinetics of the electron transfer reaction are enhanced and the overpotential is slightly lower over a wide range of c_+ (Figure S7). As expected this becomes more pronounced for small c_+ (high c_c).

When the clusters are inactive and do not sterically hinder the plating the overpotential becomes slightly lower at high c_+ and significantly higher at low c_+. This behavior represents the observations of the analysis of the Butler-Volmer rate (Figure S6). The influence of the steric hindrance dominates at high c_+, whereas the electrochemical reactivity of the clusters dominates at lower c_+. At the high current density the inactivity of the clusters starts to overcompensate the steric effects at significant higher c_+ than at the low current density.

Moreover, it can be seen that the impact of the clusters on the ion mobility in the electrolyte is insignificant at low current densities (Figure S7a), because the concentration gradient in the electrochemical cell is quite low and the local concentration of clusters and free magnesium at the electrodes is very similar to their mean concentration (Figure S9). This is in contrast to the high current density, where the rate limiting step is the transport of the electrochemically active species between the bulk and the electrode surface. In general, the limited transport leads to

* Figure S7a: The dark red points lie over the purple and dark blue ones. The light blue points are over the green ones.

higher concentration gradients in the cell. Therefore, the local electrolyte concentration $c_{\pm}$ at the electrodes are significantly lower (plating / cathode) or higher (stripping / anode) than the mean concentration, which diminishes or enhances the cluster formation respectively (Figure 2 and S9). By determining the reference potential in the middle of the electrochemical cell, the half cell potential during plating and stripping can be analyzed (Figure S8). It can be seen, that the decrease of the cell potential at high c_+ (Figure 4b region IIb) is caused by the stripping. When the local electrolyte concentration $c_{\pm}$ at the anode becomes significantly larger than the critical concentration almost all magnesium is bound in the clusters and the overpotential for stripping is maximal. This is the case for low and medium c_+ (region I and IIa). Consequently, the cell potential in this region is determined by the plating (Figure 4b and S8). Between region I and IIa the local electrolyte concentration at the cathode reaches the critical concentration (Figure 2). Consequently, for lower c_+ (region I) more ions are agglomerated, which is unfavorable for the kinetics and therefore for the overpotential. Interestingly, at low c_+ (region I) the overpotential for plating (Figure 4b and S8a) without the presence of clusters (0.1 and 0.15 M $Mg[B(hfip)_4]_2$) is significantly higher than the one of electrolyte concentrations with a high mean cluster concentration (0.45 and 0.5 M). In the latter case, the electrolyte concentration at the anode surface is around the critical concentration and therefore the local concentration of free magnesium c_+ becomes significantly higher than its mean concentration (Figure S9b), which is advantageous for the kinetics as well as the overpotential. In contrast the local c_+ at the cathode is equal or even lower than the mean concentration when there are no transport limitations (Figure S9a) or no clusters (Figure S9b) respectively, which leads to the higher overpotential for the magnesium plating.

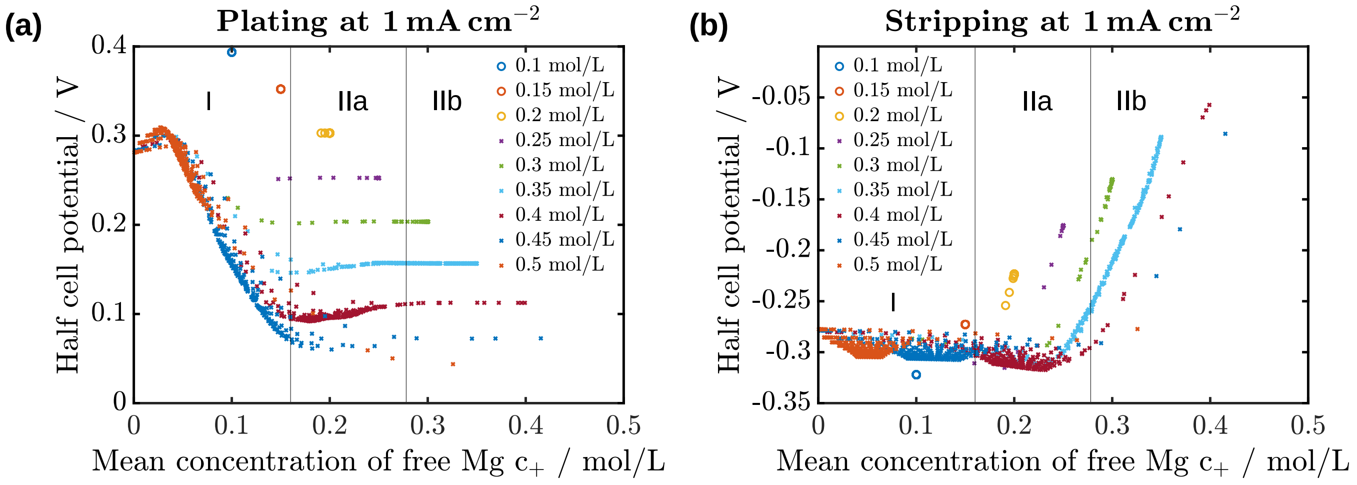

Figure S8 Parameter study: Half cell potential during plating (a) and stripping (b) at a current density of 1 mA cm^{-2}.

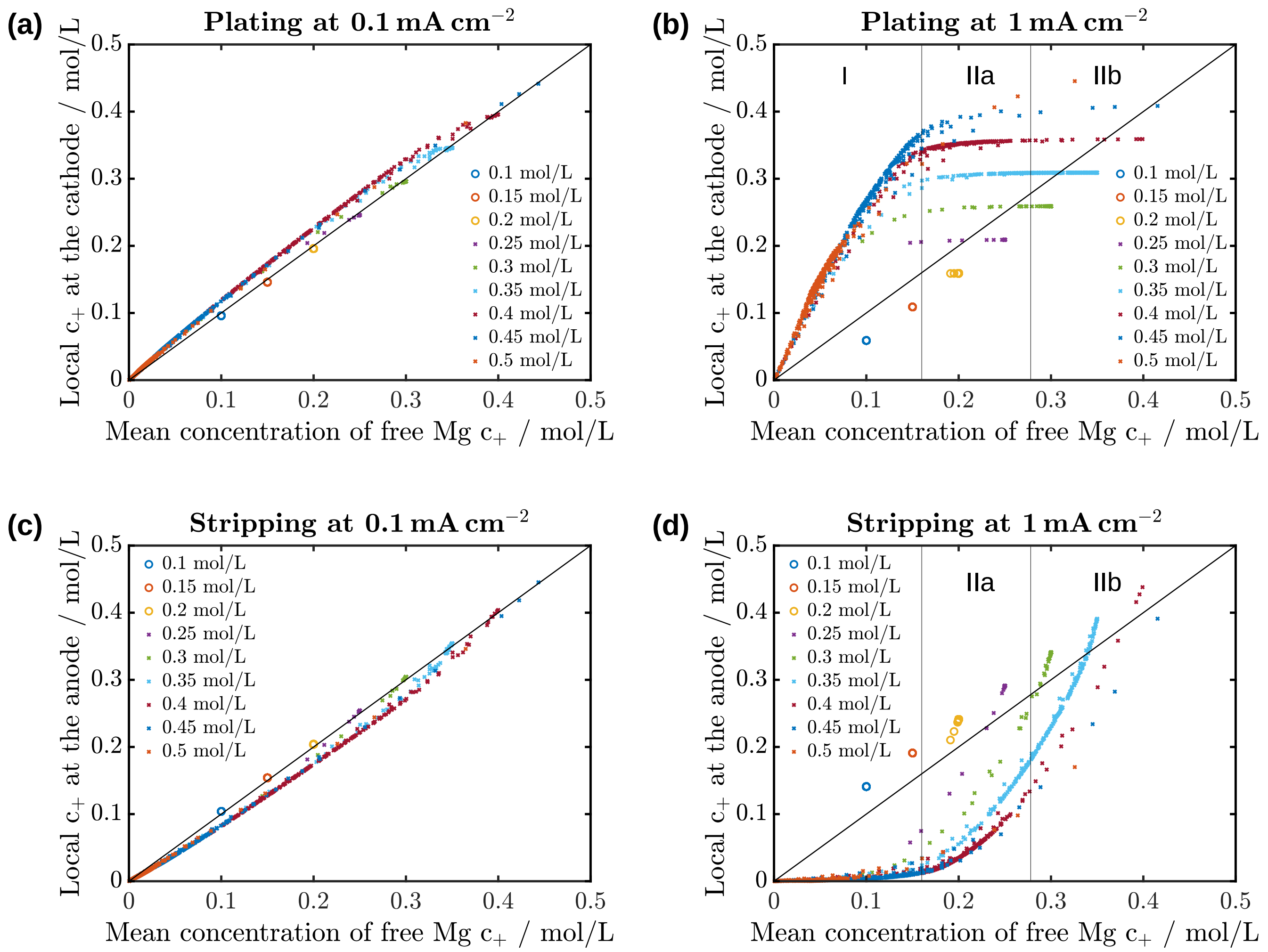

Figure S9 Parameter study: Local concentration of free magnesium c_+ during plating (top) and stripping (bottom) for current densities of 0.1 mA cm^{-2} (left) and 1 mA cm^{-2} (right).

3.3 Determination of K and z values from experimental data [3]

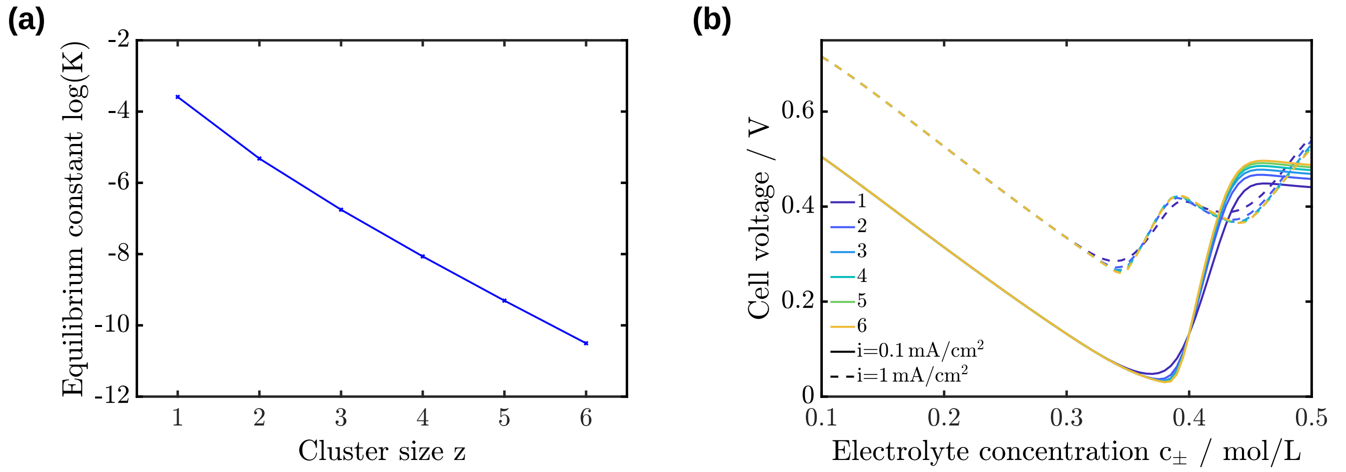

Figure S10 Parameter study: K and z values for equal overpotential of the 0.3 M and 0.4M electrolyte at the current density of 0.1 mA cm^{-2} (a) and the corresponding concentration-dependent overpotentials at the current densities of 0.1 and 1 mA cm^{-2} (b).

3.4 Sensitivity analysis for the diffusion coefficient

For a complete sensitivity analysis of the different model parameters, the impact of the diffusion coefficient D_+ was analyzed as well. Since no experimental data was available a value of $D_+ = 1 \cdot 10^{-10}\,\mathrm{m^2\,s^{-1}}$ was assumed and varied by $\pm 10\%$. Figure S11 shows, that the diffusion coefficent has only a minor impact on the qualitative and quantitative behavior of the overpotential.

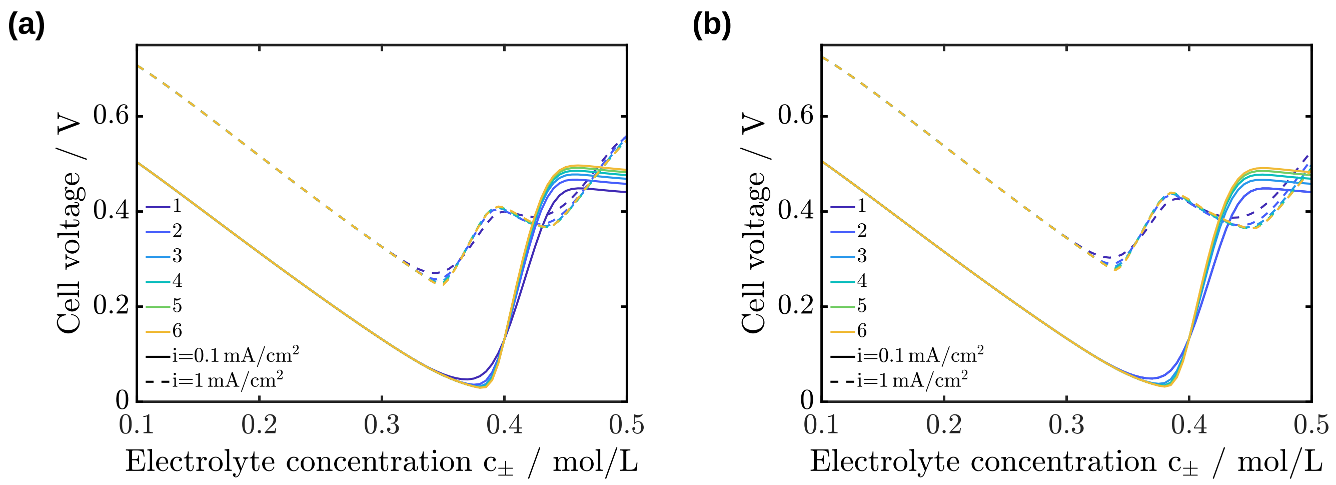

Figure S11 Parameter study: Concentration-dependent overpotentials at current densities of 0.1 and $1\,\mathrm{mA\,cm^{-2}}$ for diffuion coefficients of $D_+ = 1.1 \cdot 10^{-10}\,\mathrm{m^2\,s^{-1}}$ (a) and $D_+ = 0.9 \cdot 10^{-10}\,\mathrm{m^2\,s^{-1}}$ (b).

4 List of symbols

D	Diffusion coefficient
F	Faraday constant
I	Ionic strength
K	Equilibrium constant of cluster formation
M	Molar mass
$\vec{N}_e$	Particle flux in the bulk electrolyte
R	Universal gas constant
T	Temperature
U_0	Open circuit potential
V	Volume
a	Activity
c	Concentration
e_0	Elementary charge
f_{thermo}	Thermodynamic factor
i_{se}	Butler-Volmer flux
i_0	Scale of Butler-Volmer flux
$\vec{j}_{e,s}$	Electric current in the bulk electrolyte / bulk solid phase
k	Butler-Volmer rate constant
r	Hydrodynamic radius
r'	Radius of unsolvated ion
t_+	Transference number
w	Solvation number of Mg^{2+}

x	Solvation number of the anion
y	Number of released solvent molecules (per cluster)
$z_{+,-}$	Number of elementary charges per ion or counter ion
z	Size of the neutral cluster
$\alpha^{A,C}$	Apparent anodic and cathodic transfer coefficients
γ	Activity coefficient
ε_c	Packing density of the ions in the cluster
ε_r	Relative permittivity of the solvent
ε_0	Vacuum permittivity
η_s	Overpotential
κ	Ionic conductivity
μ	(Effective) chemical potential
ρ	Mass density
σ	Electronic conductivity
Φ_s	Electrical potential of the electrode
ϕ_e	Electrochemical potential of the electrolyte

Subscripts +, -, $\pm$, c and 0 indicate a quantity of the Mg^{2+} cation, the anion, the salt, the cluster and the solvent respectively.

References

[1] E. Samson, G. Lemaire, J. Marchand, J. J. Beaudoin, *Comput. Mater. Sci.* **1999**, *15*, 285–294.

[2] A. Latz, J. Zausch, *J. Power Sources* **2011**, *196*, 3296–3302.

[3] Z. Zhao-Karger, R. Liu, W. Dai, Z. Li, T. Diemant, B. P. Vinayan, C. Bonatto Minella, X. Yu, A. Manthiram, R. J. Behm, M. Ruben, M. Fichtner, *ACS Energy Lett.* **2018**, *3*, 2005–2013.

[4] J. Evans, C. A. Vincent, P. G. Bruce, *Polymer* **1987**, *28*, 2324–2328.
